# Validation of *Hostaalata* (Asparagaceae) as a new species and its phylogenetic affinity

**DOI:** 10.3897/phytokeys.181.64245

**Published:** 2021-09-03

**Authors:** Tetsukazu Yahara, Shun K. Hirota, Kengo Fuse, Hiroyuki Sato, Shuichiro Tagane, Yoshihisa Suyama

**Affiliations:** 1 Kyushu Open University, 744 Motooka, Fukuoka, 819-0395, Japan Kyushu Open University Fukuoka Japan; 2 Field Science Center, Graduate School of Agricultural Science, Tohoku University, 232-3 Yomogida, Naruko-onsen, Osaki, Miyagi 989-6711, Japan Tohoku University Osaki Japan; 3 The Kagoshima University Museum, Kagoshima University, 1-21-30 Korimoto, Kagoshima, 890-0065, Japan Kagoshima University Kagoshima Japan

**Keywords:** Flowering season, MIG-seq, next generation sequencing, reproductive isolation, threatened species

## Abstract

Molecular phylogenetic studies of *Hostapulchella* (Asparagaceae) and its relatives, which are native to Japan, have been conducted and resulted in a highly resolved phylogeny. Specifically, the relationship of *H.pulchella* to *H.alata* Hatusima, **nom. nud.** is investigated. These data include genome-wide SNPs obtained through conducting multiplexed ISSR genotyping by sequencing (MIG-seq). Based on these phylogenetic results, morphological observations, distribution, and differences in flowering periods of *H.alata* collections sympatric with *H.pulchella*, we find the two species closely related, but distinct. As such, we formally describe *Hostaalata***sp. nov.** from the Oita Prefecture of Kyushu island, southwestern Japan.

## Introduction

The genus *Hosta* Tratt. (Asparagaceae) is a group of 22 to 25 species endemic to East Asia and Russia (Jones 1989). The genus presents with remarkable diversity and fifteen species are endemic/native in Japan (Fujita 1976; Tamura and Fujita 2013, Tamura 2015), four in China (Chen and Boufford 2000), six in Korea (Lee et al. 2019), and one in Russia (Chen and Boufford 2000). *Hosta* are widely cultivated by gardeners in temperate areas for their attractive flowers and foliage, and continue to garner considerable interest from botanists (Jones 1989; Schmid 1991; Lee et al. 2019). Although several studies have been conducted thus far, the classification of *Hosta* remains difficult (Lee et al. 2019). Whereas Schmid (1991) enumerated 43 species in his revision of the genus, Lee et al. (2019) cited a more conservative estimate than that reported by Jones (1989) as 22 to 25 species. Recently, Lee et al. (2019) determined the sequences of the whole cpDNA genome for six Korean species and elucidated their phylogenetic relationships. Molecular phylogenetic studies are necessary for the *Hosta* species that occur in Japan, where the highest diversity has been reported (Fujita 1976; Tamura and Fujita 2013; Tamura 2015).

Here, we examined the molecular phylogeny and taxonomy of *Hosta* on Kyushu island, Japan, by focusing on a species group that contains an undescribed species, *Hostaalata* Hatusima, nom. nud. According to Arakane (2001), flowering plants of this undescribed species were discovered on Mt. Sobo on August 23, 2000, where *H.pulchella* N. Fujita is endemic. Independently, Dr. S. Hatusima examined a specimen of this species collected from Mt. Karasu-dake, 10 km NE of Mt. Sobo, and concluding that it was an undescribed species, informed Mr. Arakane of this discovery. Upon examination of the specimen, Mr. Arakane considered the *Hosta* specimen collected from Mt. Sobo to be identical to the undescribed species reported by Dr. Hatusima. Mr. Arakane then sent the Mt. Sobo specimen to Dr. Hatusima. In a letter sent to Mr. Arakane, Dr. Hatusima provided the name, *H.alata* Hatusima, nom. nud., but never formally published the species. Arakane (2001) documented the morphological characteristics of this undescribed species in Japanese, with photographs and sketched illustrations. Arakane (2001) also documented that *H.alata* blooms in late August, but *H.pulchella* blooms in July. *Hostaalata* is listed in the Red Data Book of the Oita Prefecture (Anonymous 2011); however, the name has never been validly published.

To elucidate the identity of *Hostaalata*, we visited the above-mentioned two localities, Mt. Sobo and Mt. Karasu-dake, and collected voucher specimens and silica-gel dried samples of all located *Hosta* spp. for DNA isolation. Additionally, we collected as many related species as possible during field surveys on Kyushu. From these samples, we reconstructed a phylogeny to determine their relationships. We performed multiplexed ISSR genotyping by sequencing (MIG-seq; Suyama and Matsuki 2015), which is a method for efficient reconstruction of phylogenetic relationships among closely related species (Binh et al. 2018; Strijk et al. 2020; Zhang et al. 2020). Here, we examine whether *H.alata* is a genetically distinct species from other species including *H.pulchella*. The results of our analyses support the novelty of *H.alata* and we validate the name proposed by Dr. Hatusima. Last, we discuss the implications of our findings and suggest the need to conduct further studies to elucidate the classification of *Hosta*.

## Materials and methods

### Field surveys

We visited two known localities of *Hostaalata* reported by Arakane (2001): Mt. Karasu-dake on September 24, 2020, and Mt. Sobo on September 26, 2020. Mt. Karasu-dake (683 m above sea level) is located at the end of the ridge extending northeast from Mt. Sobo (1756 m above sea level), and there is no rocky area between Mt. Sobo and Mt. Karasu-dake that is suitable for *Hostaalata* habitats. Below, the population of Mt. Karasu-dake will be referred to as *H.alata* 1, and the population of Mt. Sobo will be referred to as *H.alata* 2. We also collected H.kikutii F. Maek. var. kikutii, H.longipes (Franch. & Sav.) Matsum. var. caduca N. Fujita, *H.pulchella*, and two undescribed species (labeled *H.* sp. 1 and *H.* sp. 2) from eight localities on the main island of Kyushu, and an additional sample of H.longipesvar.caduca from the Kochi Prefecture, Shikoku Island (Table 1). A total of 29 samples collected from August to September 2020 were included in the following analysis. In addition, we collected samples of *Hosta* widely from Shikoku and Honshu, examined phylogenetic relationships, and confirmed that other Japanese species are remotely related to *Hostaalata* (data not shown).

**Table 1.** A list of samples used for phylogenetic analyses. The georeference data of the *H.alata* localities are not described to avoid facilitating collection.

Scientific name	Voucher ID	Locality	Latitude and Longitude
* H. alata *	JPN2280	Mt. Karasu-dake, Oita	Available on request
* H. alata *	JPN2281	Mt. Karasu-dake, Oita	Available on request
* H. alata *	JPN2282	Mt. Karasu-dake, Oita	Available on request
* H. alata *	JPN2283	Mt. Karasu-dake, Oita	Available on request
* H. alata *	JPN2350	Mt. Sobo, Oita	Available on request
* H. alata *	JPN2351	Mt. Sobo, Oita	Available on request
* H. alata *	JPN2372	Mt. Sobo, Oita	Available on request
H. kikutii var. kikutii	JPN1852	Kaeda Valley, Miyazaki	31.81001388, 131.3968194
H. kikutii var. kikutii	JPN1968	Mt. Osuzu, Miyazaki	32.28691111, 131.4649500
H. longipes var. caduca	JPN1248	Niyodogawa-cho, Kochi	33.47491388, 133.1060944
H. longipes var. caduca	JPN2374	Onagara, Oita	32.94940300, 131.7618470
* H. pulchella *	JPN2298	Mt. Shojidake, Miyazaki	32.81073888, 131.3482194
* H. pulchella *	JPN2306	Mt. Shojidake, Miyazaki	32.81073888, 131.3482194
* H. pulchella *	JPN2311	Mt. Shojidake, Miyazaki	32.81032222, 131.3494556
* H. pulchella *	JPN2312	Mt. Shojidake, Miyazaki	32.81032222, 131.3494556
* H. pulchella *	JPN2355	Mt. Sobo, Oita	32.83274444, 131.3520500
* H. pulchella *	JPN2356	Mt. Sobo, Miyazaki	32.82803500, 131.3467830
* H. pulchella *	JPN2360	Mt. Sobo, Miyazaki	32.82803500, 131.3467830
* H. pulchella *	JPN2364	Mt. Sobo, Miyazaki	32.82803500, 131.3467830
* H. pulchella *	JPN2365	Mt. Sobo, Miyazaki	32.82803500, 131.3467830
* H. pulchella *	JPN2368	Mt. Sobo, Oita	32.83269200, 131.3515610
* H. pulchella *	JPN2369	Mt. Sobo, Oita	32.83269200, 131.3515610
* H. pulchella *	JPN2370	Mt. Sobo, Oita	32.83269200, 131.3515610
*H.* sp. 1	JPN2012	Mt. Oninome, Miyazaki	32.70122500, 131.5138861
*H.* sp. 1	JPN2013	Mt. Oninome, Miyazaki	32.70122500, 131.5138861
*H.* sp. 1	JPN2208	Mt. Mukabaki, Miyazaki	32.62879900, 131.5790560
*H.* sp. 1	JPN2209	Mt. Mukabaki, Miyazaki	32.62879900, 131.5790560
*H.* sp. 1	JPN2210	Mt. Mukabaki, Miyazaki	32.62879900, 131.5790560
*H.* sp. 2	JPN2292	Mt. Sobo, Oita	32.82829166, 131.3904722

### DNA isolation, sequencing, and construction of SNP-based phylogenetic trees

Total DNA was extracted from dried leaves using the CTAB method (Doyle and Doyle 1990). *De novo* SNP discovery was performed using MIG-seq. Based on methods reported by Suyama and Matsuki (2015), we prepared an MIG-seq library using a two-step PCR amplification process with minor modifications; the annealing temperature of the first PCR was altered from 48 °C to 38 °C. The second-round PCR products were purified in the size range of 300–800 bp and sequenced using the Illumina MiSeq platform (Illumina, San Diego, CA, USA) and the MiSeq Reagent Kit v3 (150 cycles, Illumina). The sequencing of the first 17 bases of reads 1 and 2 (SSR primer regions and anchors) was skipped using ‘DarkCycle’. Low-quality reads and extremely short reads containing adapter sequences were removed using Trimmomatic 0.39 (Bolger et al. 2014). The Stacks 2.41 pipeline software (Catchen et al. 2013; Rochette et al. 2019) was used to obtain individual genotypes with the following parameters: minimum depth of coverage required to create a stack (*m*) = 3, maximum distance between stacks (*M*) = 2, maximum mismatches between loci when building the catalog (*n*) = 2. Three different filtering criteria were considered for quality control of the SNP data. First, any SNP site where one of two alleles had less than three counts was filtered out because it was difficult to distinguish polymorphisms from sequencing errors when the minor allele count of SNPs is too low (Roesti et al. 2012). Second, loci containing SNPs with high heterozygosity (Ho ≥ 0.6) were removed because excess heterozygosity might have resulted from artifactual loci built from several paralogous genomic regions. Third, a SNP was excluded if the number of samples shared by the SNP was below the reference value R; the minimum percentage of samples that retained a SNP. As the resolution of phylogenetic trees depends on R (Wagner et al. 2013), we used four SNP datasets in which the reference value R was changed in the following four steps: R = 0.1, 0.3, 0.5, and 0.8. As is described in Results, the basal topology of phylogenetic trees did vary with R values. The variability of topology with R values is probably derived from the limited sample sizes of *H.alata* 1, *H.alata* 2, and *H.* sp.1, ranging from 3 to 5. Under these low sample sizes, phylogenetic reconstructions using datasets with high R values tend to neglect the presence of SNPs unique to each lineage. Furthermore, phylogenetic reconstruction using datasets with low R values can contain noise with artifacts. The variability of the topology is expected to be reduced by using more samples for each lineage, but we could collect limited number of samples for *H.alata* 1, *H.alata* 2, and *H.* sp.1 because these plants were found on high vertical cliffs.

Phylogenetic trees based on SNPs was inferred using the maximum likelihood method implemented in RAxML 8.2.10 (Stamatakis 2014). We used a GTRCAT model and performed 1,000 replicates of parallelized tree search bootstrapping. Considering a possibility of reticulate relationship due to past hybridization, we also reconstructed a split network using SplitsTree4 4.14 (Huson and Bryant 2006) by implementing neighbor-net algorithm with the uncorrelated P distance matrix calculated from the SNPs matrix. We also performed an analysis of population genetic structure using Structure 2.3.4 (Pritchard et al. 2000). This software assumes a population genetic model in which there are K populations, each of which has a unique set of allele frequencies at each locus, and the individuals in the sample are probabilistically assigned to populations. If a multi-locus genotype of an individual indicates a sign of mixture, it is assigned to more than one population. To estimate the log-likelihood for each model with a different number of populations (K = 1–10), we performed 20 independent runs with a burn-in of 100,000 steps and additional 100,000 steps with the admixture model. Optimal K values were determined by using the Delta K method of Evanno et al. (2005) in Structure Harvester (Earl and Vonholdt 2012).

## Data resources

All raw MIG-seq data were deposited at the DDBJ Sequence Read Archive (DRA) with accession number DRA011465.

## Results

### A phylogenetic tree reconstructed using MIG-seq

A total of 17,666,364 raw reads (609,185 ± 16,586 reads per sample) were obtained by performing MIG-seq. After quality control, 16,924,537 reads (583,605 ± 16,419 reads per sample) were used for further analyses. After *de novo* SNP detection and filtering, the following four datasets with different R values were used: R = 0.1, 16,510 loci, 31,769 SNPs; R = 0.3, 8,163 loci, 18,296 SNPs; R = 0.5, 3,345 loci, 8,037 SNPs; and R = 0.8, 460 loci, 1,069 SNPs.

**Figure 1.**
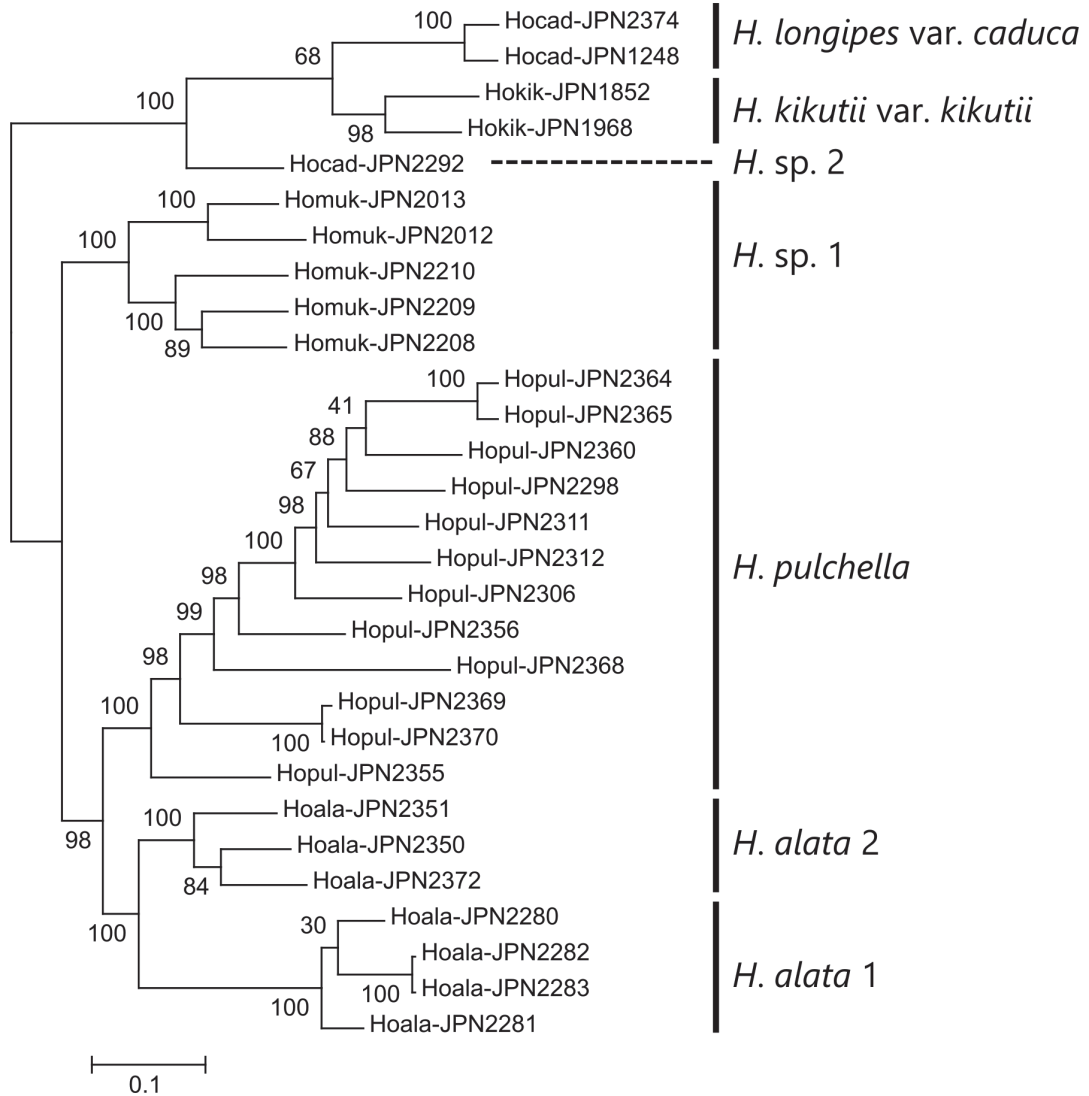
A molecular phylogeny of *Hostaalata* and its relatives reconstructed on the basis of MIG-seq data. Bootstrap values are shown on the internodes. The scale bar represents the average number of substitutions per SNP site.

A phylogenetic tree reconstructed using MIG-seq by setting R = 0.5 (Fig. 1; SNPs shared among 15 samples of the 29 total were used) recovered *Hostaalata* as sister to *H.pulchella* (98% bs). The two known populations of *H.alata*, Karasu-dake (*H.alata* 1: JPN2281–2283) and Sobo (*H.alata* 2: JPN2350, JPN2351, JPN2372) were each recovered monophyletic (100 bs), and these form a well-supported clade (100 bs). Three samples from Mt. Mukabaki (JPN2208–2210) and two samples from Mt. Oninome (JPN2012, 2013) formed a clade supported by a 100% bootstrap value, designated as *H.* sp. 1 (Fig. 1). The clade that included *H.alata*, *H.pulchella*, and *H.* sp. 1 (100 bs) was sister to the remaining sampled species including *H.kikutii*, H.longipesvar.caduca, and a sample from Mt. Sobo designated as *H.* sp. 2 (100 bs).

**Figure 2.**
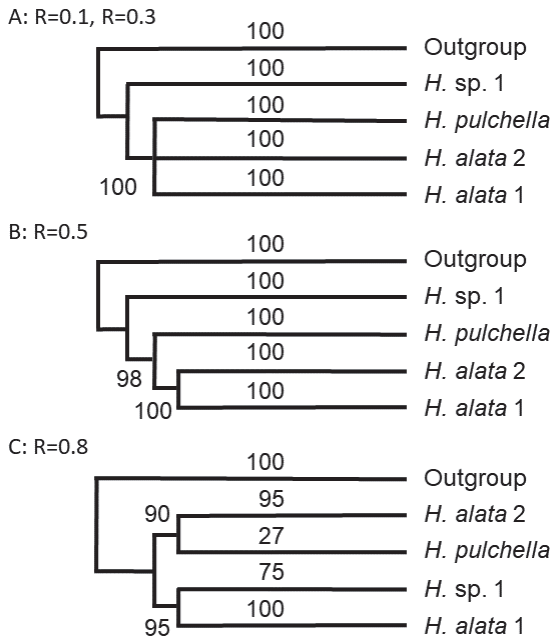
Three different topologies of *Hosta* phylogenetic trees reconstructed on the basis of MIG-seq conducted using different settings for the proportion of missing loci, R **A** the topology when R = 10 or 30 **B** the topology when R = 50 **C** the topology when R = 80. Bootstrap values are shown on the internodes.

The topology of the phylogenetic reconstructions using MIG-seq data varied with the setting of R, the minimum percentage of samples that shared a SNP (Fig. 2). When R = 0.1 (SNPs shared by three or more samples are used; Fig. 2A), or R = 0.3 (SNPs shared by nine or more samples are used; Fig. 2A), *Hostaalata* 1, *H.alata* 2, and *H.pulchella* were trichotomous, and the monophyly of the clade including these three was supported by a 100% bootstrap value; *H.* sp. 1 was sister to this clade. When R = 0.5 (SNPs shared by 15 or more samples were used; Fig. 2B), the two *H.alata* populations were sister to *H.pulchella*. When R = 0.8 (SNPs shared by 24 or more samples are used; Fig. 2C), *H.alata* 1 and *H.alata* 2 were not monophyletic; *H.alata* 1 was sister to *H.* sp. 1 and *H.alata* 2 was sister to *H.pulchella* (Fig. 2C).

### A split network reconstructed using MIG-seq

**Figure 3.**
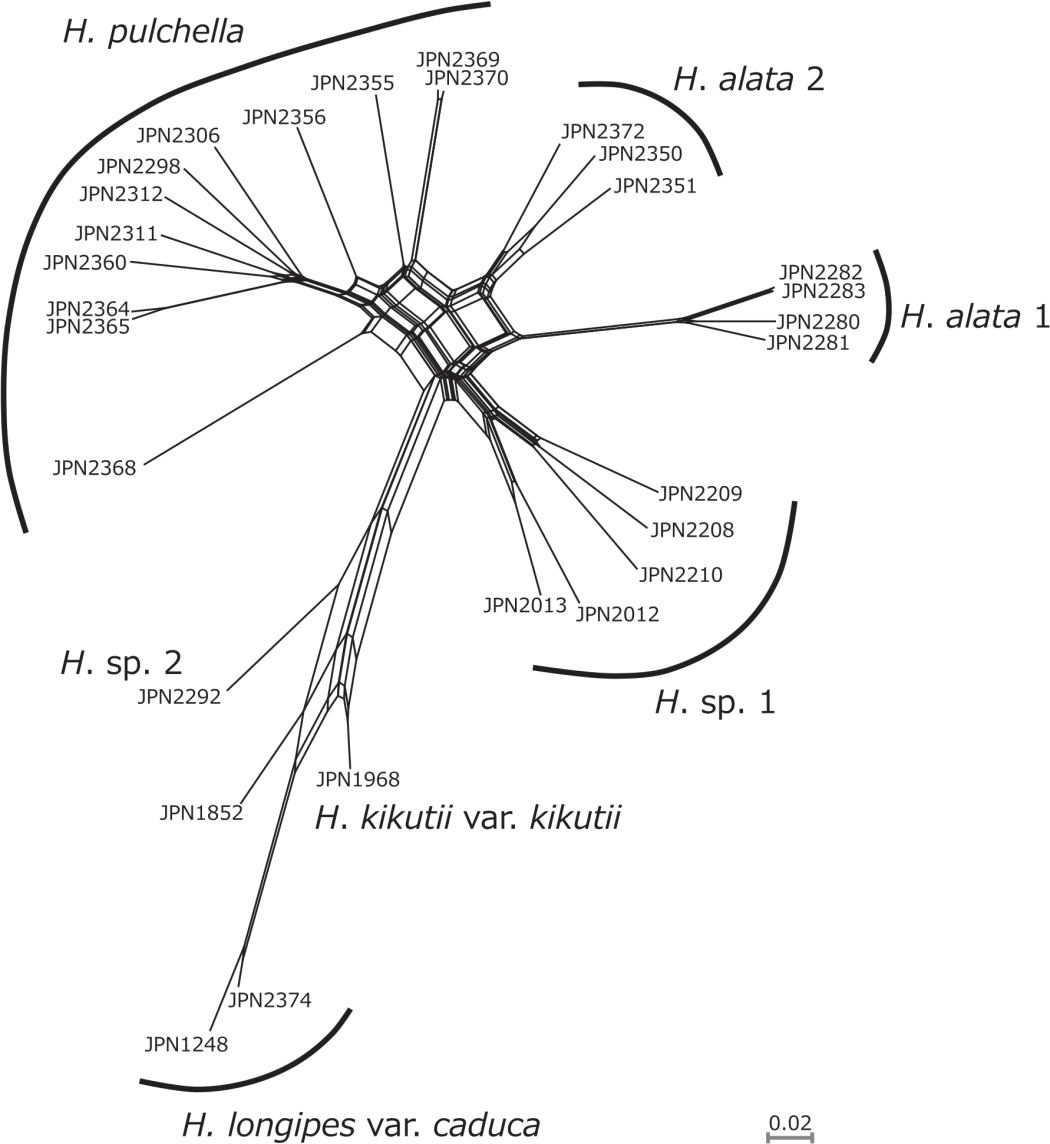
A split network of *Hostaalata* and its relatives reconstructed on the basis of MIG-seq data by setting R = 0.5.

A split network reconstructed using R = 0.5 (Fig. 3) showed that *Hostapulchella*, *H.alata* 1, *H.alata* 2, and *H.* sp. 1 formed four distinct clusters. *Hostaalata* 2 was placed between *H.pulchella* and *H.alata* 1. Two samples of H.longipesvar.caduca were clustered together, but nested within H.kikutiivar.kikutii. *Hosta* sp. 2 was placed outside the cluster including H.longipesvar.caduca and H.kikutiivar.kikutii.

### STRUCTURE analysis

**Figure 4.**
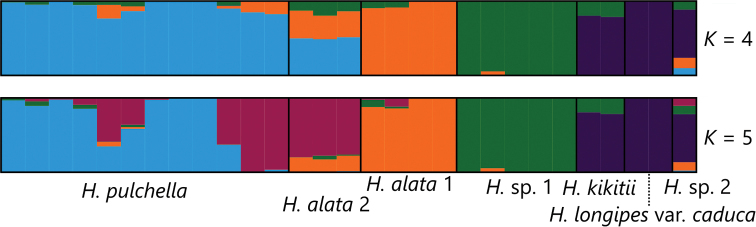
Population genetic structure of *Hostaalata* and its relatives at K = 4 and 5 inferred from MIG-seq data by setting R = 0.5.

In the Structure analysis, using R = 0.5, K = 4 or 5 was optimal: delta K increased from K = 3 to K = 4, took almost the same value at K = 4 and K = 5, and decreased from K = 5 to K = 6. When K = 4 (Fig. 4), *H.pulchella*, *H.alata* 1, *H.* sp. 1, and a group including H.kikutiivar.kikutii, H.longipesvar.caduca represented genetically unique populations, and *H.alata* 2 showed a mixture of genetic elements derived from *H.pulchella* and *H.alata* 1. Genetic elements from *H.* sp. 1 were found in three other genetically unique populations, albeit infrequently. When K = 5, *H.alata* 2 and some individuals of *H.pulchella* represented the fifth genetically unique populations, and thus *H.pulchella* was shown to be genetically heterogeneous. *Hostaalata* 2 had some genetic elements derived from *H.alata* 1.

**Figure 5.**
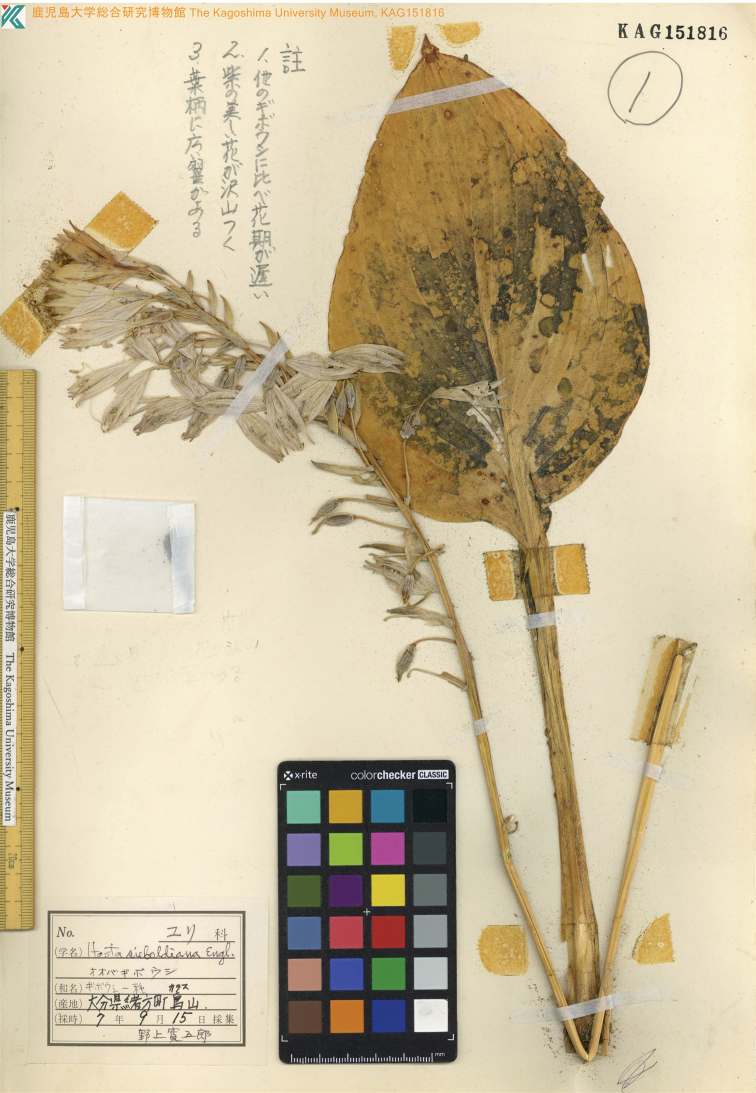
Holotype of *Hostaalata* Hatusima ex Yahara. (*Nogami s.n.* KAG151816, available from https://dbs.kaum.kagoshima-u.ac.jp/musedb/s_plant/picture/KAG151816/KAG151816.jpg).

### Field observations

On Mt. Karasu-dake, a small population of *Hostaalata* 1 is found on cliffs along the ridge line at 676 m elevation. On Mt. Sobo, *H.alata* 2 grows along the ridge line at 1500 m elevation, where we also found a population of *H.pulchella*. While *H.alata* 2 grows on steep cliffs, *H.pulchella* grows in crevices of rocks along the mountain path. In the vicinity of the peak of Mt. Sobo at 1756 m elevation, we found only *H.pulchella* growing in rock crevices. We found several (fewer than 10) flowering *H.alata* 1 in Mt. Karasu-dake on September 24, 2020, but all *H.alata* 2 plants observed in Mt. Sobo on September 26, 2020, were fruiting (fewer than 10) or sterile (ca. 20). In contrast, we collected a few flowering *H.pulchella* specimens at the peak of Mt. Shojidake, located 2 km south of Mt. Sobo, on September 25, 2020; however, other plants observed in Mt. Shojidake were fruiting or sterile.

**Figure 6.**
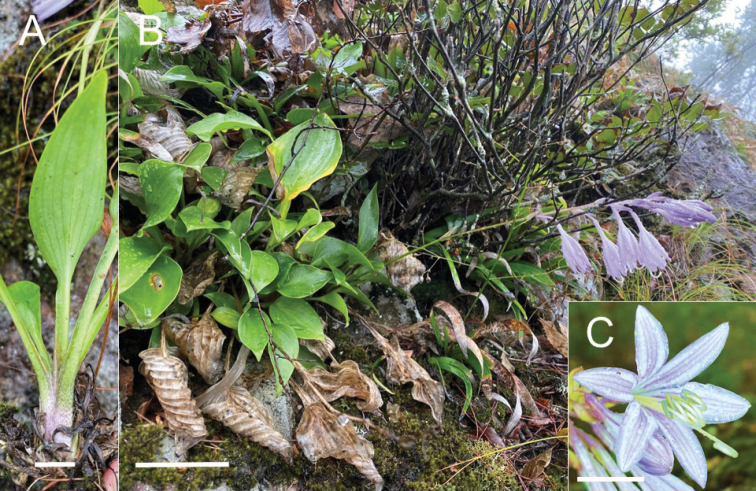
Depiction of *Hostaalata* Hatusima ex Yahara in its natural habitat in Mt. Karasu-dake, Oita Pref., Japan. **A** leaves with winged petioles **B** flowering plant **C** front view of a flower. Scale bars: 2 cm (**A**); 10 cm (**B**); 1 cm (**C**). Photographs taken by K. Fuse on September 24, 2020.

According to the photographs, sketched illustrations, and description of *H.alata* 1 (Arakane 2001) and our own observations of *H.alata* 1 and *H.alata* 2, *Hostaalata* plants in Mt. Karasu-dake and Mt. Sobo were indistinguishable in both floral and vegetative traits. *Hostaalata* was similar to *H.pulchella*, as evidenced by the presence of dark purple veins inside perianth lobes (Fig. 6C); bracts in anthesis are vivid (not withering) and erect or diagonally spreading, and leaf blades are ovate or oblong-ovate with smooth veins on the lower surface. However, *H.alata* was distinguished from *H.pulchella* by the presence of more leaves (5–9 vs. 3–4), larger leaf blades (8.5–24.5 cm long vs. 2.7–8.0(–8.9) cm long), more lateral veins (5–9 pairs vs. 3–4 pairs), wider-winged petioles (0.4–1.4 cm wide vs. 0.2–0.4 cm wide), more flowers with 9–40 flowers per scape vs. 3–4 flowers, longer pedicels (1.1–2.3 cm long vs. 0.5–0.8 cm long), and fertile bracts which are purplish green in color (vs. pale green).

On Mt. Mukabaki and Mt. Oninome, plants of *Hosta* sp. 1 were found on high vertical cliffs. When we visited these localities late September, all plants we observed were sterile. These plants were similar to *H.alata* in the number of leaves, leaf size, the number of lateral veins, and width of petiole wings.

## Discussion

The MIG-seq tree showed that *Hostaalata* was closely related to *H.pulchella* and *H.* sp. 1; the monophyly of a clade including these three species was strongly supported irrespective of R values (Fig. 2). The MIG-seq trees also showed that *H.alata* 1, *H.alata* 2, *H.pulchella*, and *H.* sp. 1 were clearly differentiated irrespective of R values. The monophyly of *H.alata* 1 and *H.alata* 2 was supported by the MIG-seq tree with R = 0.5, but not supported by the MIG-seq tree with R = 0.8. Morphologically, the former result is supported: *H.alata* 1 and *H.alata* 2 are identical in key characters and clearly distinguished from *H.pulchella*, even though the two *H.alata* populations are geographically isolated from one another. *Hostaalata* and *H.pulchella* both grow approximately at 1500 m elevation on Mt. Sobo and prefer different habitats: *H.alata* with a larger plant size prefers steep, often vertical cliffs, but *H.pulchella* owing to its smaller size grows in rock crevices along the mountain path. According to our unpublished observation, the difference in plant size is preserved even in cultivation. We did not observe any intermediate between *H.alata* 2 and *H.pulchella*. Based on this evidence, *H.alata* and *H.pulchella* are considered reproductively isolated species. The differences in flowering times between *H.alata* and *H.pulchella* may contribute to their reproductive isolation and their molecular divergence from one another. On Mt. Sobo, *H.alata* flowers from late August to late September, while *H.pulchella* flowers mostly in July (Arakane 2001). It was unusual that we collected a *H.pulchella* in flower in late September among fruiting individuals.

Split network (Fig. 3) also showed that *Hostapulchella*, *H.alata* 1, *H.alata* 2, and *H.* sp. 1 were clearly differentiated. However, *Hostaalata* 2 was placed between *H.pulchella* and *H.alata* 1, suggesting that *H.alata* 2 might be of hybrid origin between *H.pulchella* and *H.alata* 1. The K = 4 result of Structure analysis (Fig. 4) also suggested that *H.alata* 2 has genetic elements from not only *H.alata* 1 but also *H.pulchella*. However, the K = 5 result of Structure analysis showed that *H.alata* 2 and some individuals of *H.pulchella* represented the fifth genetically unique population, suggesting that these individuals of *H.alata* 2 and *H.pulchella* shared genetic elements of old origin. These findings suggest that *H.alata* 2 was differentiated from *H.alata* 1 due to geographical isolation over a long geological time, hybridized with *H.pulchella* in the past, but retained its morphological traits that characterize *H.alata* today. Because *H.alata* 2 is morphologically distinct from *H.pulchella*, flowering from late August to late September when *H.pulchella* is fruiting, and *H.alata* 2 is separated from *H.pulchella* in the split network (Fig. 3), we taxonomically identify *H.alata* 2 as a population of *H.alata*. It is now widely known that a taxonomic species often has a history of past hybridization (Suarez-Gonzalez et al. 2018), and *H.alata* may be one example of such species. The split network (Fig. 3) also suggested that H.longipesvar.caduca and H.kikutiivar.kikutii may have a history of hybridization, because two species were nested despite their morphological distinction.

In the MIG-seq tree constructed in the present study, *Hosta* sp. 1 collected from Mt. Mukabaki and Mt. Oninome formed a distinct clade supported by a 100% bootstrap value. In the split network, *Hosta* sp. 1 formed a distinct cluster outside of *H.alata* and *H.pulchella*. These findings suggest that *Hosta* sp. 1 is another undescribed species. However, only sterile plants in this clade were collected. Further studies on flowering materials are warranted to describe this clade as a species.

It is likely that *H.* sp. 2 is another undescribed species. To better characterize *H.* sp. 2, however, further studies on two polymorphic species, *H.kikutii* and *H.longipes*, are needed. Notably, Hostakikutiivar.kikutii, with vivid bracts in anthesis, is shown to be sister to H.longipesvar.caduca with withering bracts in anthesis. The condition of fertile bracts has been emphasized as a discriminating trait in the taxonomy of *Hosta* (Maekawa 1940; Fujita 1976; Tamura and Fujita 2013; Tamura 2015). Consequently, *Hostalongipes* is placed in Section Picnolepis (Maekawa 1940; Fujita 1976; Zonneveld and Van Iren 2001), whereas *H.kikutii* is placed in Section Rhynchophorae (Maekawa 1940; Zonneveld and Van Iren 2001) or Section Helipteroides (Fujita 1976). Further studies are needed to understand how the condition of fertile bracts varies between closely related species. By examining the phylogenetic relationships of more species using MIG-seq, significant developments are expected in the taxonomic revision of *Hosta* species.

## Taxonomy

### Hosta alata

Taxon classificationPlantae

Hatus. ex Yahara sp. nov.

EC77E143-2659-51CA-9BB8-17C6C64641F8

urn:lsid:ipni.org:names:77219558-1

Figs 5 , 6 Japanese name: Bungo-giboshi

#### Diagnosis.

*Hostaalata* is distinguished from *H.pulchella* by the presence of more leaves (5–9 vs. 3–4), larger leaf blades (8.5–24.5 cm long vs. 2.7–8.0(–8.9) cm long), more lateral veins (5–9 pairs vs. 3–4 pairs), wider-winged petioles (0.4–1.4 cm wide vs. 0.2–0.4 cm wide), more flowers (9–40 flowers per scape vs. 3–4 flowers), longer pedicels (1.1–2.3 cm long vs. 0.5–0.8 cm long), and fertile bracts which are purplish green in color (vs. pale green).

#### Type.

Japan. Oita Pref.: Ogata-cho, Mt. Karasu-dake (recorded as Karasu-yama), September 15, 1995, with flowers, *K. Nogami s.n.* (***holotype***: KAG 151816!).

#### Description.

Herbs perennial, up to 48 cm in height, including scape. Plants green (not whitish-green). Leaves basal, spiral, long petiolate, 5–9 per ramet; blades ovate or oblong-ovate, 8.5–20.5 × 3.7–15.0 cm, thinly papery, glabrous on both surfaces, base cuneate to subcordate, apex acute to short acuminate, acumen to 1 cm long, margin entire, veins in 5–9 pairs, smooth on the lower surface; petioles 6.0–15.5 cm long, 0.4–1.4 cm wide, winged, wings 0.5–2.0 mm wide, glabrous, reddish maculate proximally. Scape 20–48 cm, terete. Raceme 9–40-flowered; sterile bracts at low to middle part of rachis 2, longer than 3 cm, apex not seen (broken and disappeared); one fertile bract subtending each flower, vivid (not withering) in anthesis, erect or diagonally, purplish green, oblong-lanceolate, boat-shaped, 0.8–2.4 × 0.2–0.6 cm, membranous, glabrous, apex acuminate. Flowers not fragrant, 4.2–7.4 cm long; pedicels 1.1–2.3 cm long, glabrous. Perianth light purple, funnel-form, 3.0–5.4 cm long, glabrous, 6-lobed; tube ca. 0.2 cm wide at base, abruptly dilated from apical 2/3, to 0.7–1.4 cm wide at throat, lobes narrowly triangular, 0.9–1.2 cm long, apex acute. Stamens 6, slightly shorter than perianth, not exserted; filaments white, free, 3.4–4.2 cm long, glabrous, anthers yellow, 2.5–3.5 mm long. Ovary oblong-ellipsoid, ca. 8 mm long, glabrous, style 3.7–4.5 cm long, upwardly curved at the distal part, subequal to 0.2–0.3 cm exserted from perianth, glabrous, stigma capitate. Capsule dark purple, dotted, cylindrical, 1.7–2.3 × 0.3–0.5 cm, 3-angled.

#### Phenology.

Flowering from late August to late September, and fruiting in late September and probably to October.

#### Distribution and habitat.

Oita Prefecture, Japan (endemic). This species grows on rock cliffs in the southern part of the Oita Prefecture on the main island of Kyushu.

#### Etymology.

The specific epithet is derived from its winged petioles.

#### Conservation status.

This species is listed in the Red Data Book Oita (Anonymous 2011) as Endangered (EN). The total number of fertile individuals in the two localities was estimated to range between 50 and 100. While the population of Mt. Sobo is located in the protected area of the Sobo Katamuki National (Kokutei) Park, the population occurring in Mt. Karasu-dake is not protected. It grows on steep rocky cliffs and the localities are not commonly known, and thus the populations appear stable. However, the formal description of this species may increase the collection pressure. Protection measures are currently being planned with administration.

#### Additional specimens examined.

Japan. Oita Pref.: Ogata-cho, Mt. Karasu-dake, on cliff, 676 m elevation, September 24, 2020, with flowers, *T. Yahara* et al. *JPN2280* -2283(FU!); Ogata-cho, Ogouchi Forest Road, 780 m elevation [in the vicinity of the *JPN2280* - 2283 collection site at Mt. Karasu-dake], September 19, 2001, photographs taken by M. Arakane (KAG 151818!); Mt. Sobo, August 23, 2000, with flowers, *M. Arakane AR-43465* (KAG 151817!); Mt. Sobo, September 26, 2020, sterile, *T. Yahara* et al. *JPN2350*, *2351*, *2372* (FU!).

## Supplementary Material

XML Treatment for Hosta alata
